# Advances in Targeted and Immunotherapy for High-Risk Cutaneous Malignancies

**DOI:** 10.32604/or.2025.073383

**Published:** 2026-02-24

**Authors:** Amy J. Petty, Drew A. Emge, Adela R. Cardones

**Affiliations:** Division of Dermatology, Department of Internal Medicine, University of Kansas Medical Center, Kansas City, KS 66103, USA

**Keywords:** Cutaneous malignancy, squamous cell carcinoma, basal cell carcinoma, melanoma, immunotherapy

## Abstract

Skin cancer remains the most commonly diagnosed malignancy worldwide, with basal cell carcinoma (BCC), cutaneous squamous cell carcinoma (cSCC), and melanoma representing the most clinically significant types. While traditional treatments are effective for early-stage disease, advanced or metastatic cases often pose significant therapeutic challenges. Patients with high-risk or recurrent disease face limited options and poor prognoses. The emergence of immunotherapy has dramatically transformed the treatment landscape across multiple cancer types, including cutaneous malignancies. This review highlights recent advancements in immunotherapeutic strategies for BCC, cSCC, and melanoma, underscoring their growing importance in dermatologic oncology. We synthesize current evidence and ongoing clinical trials for immunotherapy across these three skin cancer types. We also explore the molecular mechanisms underpinning immune responsiveness and potential biomarkers of response. As immunotherapy continues to expand within dermatology, understanding its role, limitations, and future directions is essential for optimizing patient care. The integration of immunotherapy into dermatologic practice represents not only a therapeutic innovation but also a shift toward precision medicine in cutaneous oncology.

## 1 Introduction

Non-melanoma skin cancers (NMSCs), primarily basal cell carcinoma (BCC) and cutaneous squamous cell carcinoma (cSCC), constitute the majority of skin malignancies, with BCC being the most common cancer worldwide and cSCC as the second most frequent [1–3]. Although melanoma accounts for a minority of skin cancer cases, it is responsible for the majority of skin cancer-related mortality due to its aggressive clinical course and metastatic capability [4]. The global incidence of all three skin cancer types continues to rise steadily, influenced by cumulative ultraviolet (UV) exposure, population aging, and increased awareness and detection efforts [5].

Historically, surgical excision remained the cornerstone of management for localized skin cancers and continues to offer excellent cure rates in early disease [6]. However, the recognition of high-risk clinical and pathological features in cSCC and the emergence of advanced, treatment-resistant forms of BCC have highlighted the need for systemic therapies beyond surgery and radiotherapy [7,8]. Simultaneously, melanoma has pioneered modern cancer immunotherapy, demonstrating unprecedented survival gains with immune checkpoint blockade targeting cytotoxic T-lymphocyte antigen 4 (CTLA-4) and programmed death-1 (PD-1) pathways [9].

The molecular pathogenesis of skin cancers reflects a complex interplay between environmental carcinogens, particularly UV-induced DNA damage, and intrinsic genetic and immunologic vulnerabilities [10,11]. BCC and cSCC, collectively referred to as nonmelanoma skin cancers, share common risk factors but diverge in their molecular pathways—BCC being driven largely by Hedgehog signaling and cSCC by mutations in tumor protein 53 (TP53) and dysregulated epidermal growth factor receptor (EGFR) pathways [12,13]. In contrast, melanoma arises from melanocytes and is marked by a high mutational burden and marked immunogenicity, rendering it especially amenable to immune modulation [14].

In this review, we provide an in-depth analysis of the pathogenesis, immune landscape, current Food and Drug Administration (FDA)-approved therapies, and clinical trials for BCC, cSCC, and melanoma. This review focuses on key agents such as Hedgehog inhibitors (vismodegib, sonidegib), immune checkpoint inhibitors/ICIs (cemiplimab, pembrolizumab, nivolumab), and emerging dual ICIs (nivolumab-relatlimab). We discuss potential biomarkers including PD-L1 expression, tumor mutational burden (TMB), and interferon-gamma (IFN-γ)–related signatures, as well as challenges such as immune-related adverse events and resistance mechanisms. We aim to highlight recent scientific advances, inform therapeutic decision-making, and identify gaps in knowledge that may guide future research.

## 2 Basal Cell Carcinoma

### 2.1 Pathogenesis

Basal cell carcinoma (BCC) originates from the basal cells of the epidermis or follicular stem cells and is strongly associated with chronic UV radiation exposure, particularly ultraviolet B (UVB) [15]. UV-induced DNA damage, specifically the formation of pyrimidine dimers, is a key mutagenic mechanism that initiates oncogenesis [16]. UVB radiation causes covalent bonding between adjacent pyrimidine bases, producing cyclobutane pyrimidine dimers (CPDs) and 6-4 photoproducts, which distort the DNA helix and disrupt normal base pairing. If these lesions are not corrected by nucleotide excision repair, they lead to characteristic UV signature mutations, particularly cytosine-to-thymine (C→T) or tandem CC→TT transitions at dipyrimidine sites [17]. These mutations frequently occur in tumor suppressor genes such as TP53, impairing cell cycle checkpoints and apoptosis, thereby permitting clonal expansion of mutated keratinocytes [15]. In addition, chronic UV exposure induces mutations in components of the Hedgehog signaling pathway, particularly Patched 1 (PTCH1) and Smoothened (SMO), which are central drivers of BCC pathogenesis [18]. Beyond direct DNA mutagenesis, UV radiation also promotes oxidative stress, immunosuppression, and local inflammation, which synergize to facilitate tumor initiation and progression.

The principal molecular driver of BCC is the aberrant activation of the Hedgehog (HH) signaling pathway [19]. Under normal physiological conditions, the HH pathway is inactive due to inhibition of the SMO receptor by PTCH1. In BCC, inactivating mutations in PTCH1 or activating mutations in SMO disrupt this inhibition, resulting in constitutive activation of downstream transcription factors such as Glioma-associated oncogene homolog 1 (GLI1) and Glioma-associated oncogene homolog 2 (GLI2), which promote cell proliferation and survival. PTCH1 loss-of-function mutations are the most common alterations, occurring in more than 70% of sporadic BCCs, while activating mutations in SMO account for an additional subset of cases [20]. The consequence is unchecked transcription of target genes that regulate proliferation, anti-apoptotic signaling, and angiogenesis. Moreover, germline mutations in PTCH1 underlie nevoid basal cell carcinoma syndrome (Gorlin syndrome), a disorder characterized by multiple early-onset BCCs, further underscoring the pivotal role of Hedgehog signaling dysregulation in tumorigenesis [21]. In addition to PTCH1 and SMO, mutations in Suppressor of Fused (SUFU)—a critical downstream negative regulator that normally sequesters GLI proteins in the cytoplasm—can result in their constitutive nuclear localization and transcriptional activity, bypassing upstream control mechanisms [22]. Although SUFU mutations are less frequent than PTCH1 or SMO alterations, they are strongly associated with hereditary BCC predisposition and aggressive tumor phenotypes [23]. Similarly, amplification of GLI transcription factors, particularly GLI1 and GLI2, further enhances pathway output by directly increasing transcriptional drive of genes regulating cell cycle progression, epithelial-to-mesenchymal transition, and resistance to apoptosis [24]. These additional layers of dysregulation emphasize that HH pathway activation in BCC is not limited to receptor-level mutations but extends to downstream effectors, amplifying oncogenic signaling and complicating therapeutic targeting.

In addition to HH pathway dysregulation, other genetic and epigenetic events contribute to BCC pathogenesis. Loss-of-function mutations in tumor suppressor genes such as TP53 and epigenetic promoter hypermethylation of Ras association domain family member 1 (RASSF1A), Decoy receptor 1 (DCR1), Decoy receptor 2 (DCR2) and Adenamtous polyposis coli (APC) have been reported [25,26]. Furthermore, dysregulation of genes in the tumor microenvironment of BCC such as Gremlin 1 (GREM1), which is a BMP antagonist, and interleukin-6 (IL-6) signaling pathways has also been implicated, indicating a broader network of tumor-promoting mechanisms [27,28].

While BCC is characterized by a high mutational burden due to cumulative UV damage, its biological behavior is generally indolent, with a very low propensity for metastasis (<0.1%) [29]. However, in rare cases, locally advanced BCC (laBCC) or metastatic BCC (mBCC) may develop, often requiring systemic therapy targeting critical driver mutations.

### 2.2 Immune Landscape of Basal Cell Carcinoma

The immune microenvironment of BCC is characterized by a paradox: despite its high mutational burden and associated neoantigen load—features that typically promote immune recognition—BCCs frequently evade immune surveillance and progress locally [30]. This immune evasion is facilitated by both tumor-intrinsic and microenvironmental mechanisms.

A hallmark of the BCC immune microenvironment is the suppression of antigen presentation [31]. BCC tumors exhibit downregulation of major histocompatibility complex class I (MHC-I) molecules, particularly β2-microglobulin, a key component of MHC-I, which diminishes CD8^+^ T-cell recognition of tumor antigens [32]. Lower class 1 antigen expression in BCC is highly correlated with overall decreased tumor differentiation and more aggressive tumor phenotype [30]. This is why treatment with imiquimod, which activates toll-like receptors (TLR) 7 and 8 and downstream MHC 1 expression, can be utilized to treat BCC [33]. Additionally, BCC tumor cells and tumor microenvironment contain higher levels of Th2 cytokines such as IL-4, IL-5, IL-10, IL-13 and transforming growth factor-beta (TGF-β), fostering an immunosuppressive milieu [34,35]. In addition to cytokines, regulatory T cells (Tregs) and Th2-skewed responses dominate the immune infiltrate in BCC [30]. Tregs limit effective anti-tumor immune responses by suppressing cytotoxic T-cell activity, while Th2 cytokines, including IL-4 and IL-13, contribute to tumor-promoting inflammation [34,36]. Beksaç et al. also identified a predominance of immunosuppressive M2 macrophages in BCC though no association of M2 macrophage level with recurrence was identified [37]. Lastly, elevated levels of IL-17, IL-22, and IL-23 have been implicated in promoting keratinocyte proliferation and sustaining tumor growth in BCC [38]. Though exact mechanism remains elusive, exposure of BCC cell lines to IL-22 results in increased phosphorylation of the Signal transducer and activator of transcription 3 (STAT3) and Mitogen-activated protein kinase (MAPK) pathways and constitutive p65 phosphorylation, a proxy of Nuclear factor kappa-light chain-enhancer of activated B cells (NF-κB) signaling, was identified following IL-17 exposure [39].

Cancer-associated fibroblasts (CAFs) are another prominent feature of the BCC tumor microenvironment. These stromal cells secrete chemokines such as CXCL12 and CCL17, which attract immunosuppressive cells and further restrict anti-tumor immunity [28]. CAFs also produce matrix metalloproteinases (MMPs), particularly MMP-2 and MMP-9, which remodel the extracellular matrix and facilitate tumor invasion [40]. Beyond these functions, CAFs secrete growth factors including TGF-β, vascular endothelial growth factor (VEGF), and fibroblast growth factor (FGF), which promote angiogenesis, epithelial-mesenchymal transition, and survival of malignant keratinocytes [41,42].

Importantly, immune checkpoint pathways, especially programmed death-1 (PD-1) and its ligand PD-L1, are upregulated in BCC [43]. Chang et al. quantified PD-L1 staining in BCCs with a positive cutoff of ≥5% and reported 89.9% of tumors stained positive for this antigen. Additionally, treatment-naïve tumors presented with lower intensity of PD-L1 staining compared to tumors previously treated with various treatments [44]. This provides a compelling rationale for the use of immune checkpoint inhibitors in advanced disease settings of BCC.

Overall, while BCC is immunogenic at the molecular level, a highly suppressive microenvironment dominated by Tregs, cytokine signaling, and impaired antigen presentation limits effective anti-tumor immunity. Recent therapeutic strategies are increasingly focused on reversing this suppression through immune checkpoint blockade and other immune-modulatory interventions.

### 2.3 FDA-Approved Therapies for Basal Cell Carcinoma

The therapeutic landscape of BCC has evolved significantly in the past decade, especially for patients with laBCC or mBCC who are not candidates for curative surgery or radiotherapy. The United States FDA has approved two main classes of systemic therapies for advanced BCC: Hedgehog pathway inhibitors (HHIs) and immune checkpoint inhibitors (ICIs). HHIs are first line treatment for laBCCs per the NCCN guidelines [45].

#### 2.3.1 Hedgehog Pathway Inhibitors (HHIs)

Given that aberrant Hedgehog signaling is the key oncogenic driver in BCC, targeting this pathway has been a major therapeutic breakthrough. Two oral HHIs have received FDA approval—Vismodegib and Sonidegib.

Vismodegib was the first HHI approved by the FDA in 2012 and works as a selective SMO inhibitor that blocks Hedgehog pathway activation. It is indicated for laBCC and mBCC [46]. The landmark ERIVANCE trial, which evaluated vismodegib, was an international, single-arm study consisting of two cohorts that included 71 patients with laBCC and 33 patients with mBCC, all treated with a 150 mg dose [46,47]. In this study, the objective response rate (ORR) was 43% among patients with laBCC, with 21% achieving a complete response [46,47]. At the 30-month follow-up, 60.3% of patients demonstrated an ORR, and the median duration of response reached 26.2 months [48]. Complementing these findings, the SafeTy Events in VismodEgiv (STEVIE) trial—an open-label, multicenter study enrolling 1215 patients—reported an ORR of 68.5% for laBCC, with a median response duration of 23 months [49]. More recently, a 2023 meta-analysis pooling data across multiple vismodegib studies found an ORR of 74.2% for laBCC and 41.9% for mBCC [50].

Following the landmark studies, several recent international studies also have demonstrated favorable efficacy of vismodegib. In Slovenia, a retrospective study of 39 patients with laBCC and multiple BCCs reported an ORR of 80%, with a median treatment duration of 9.9 months [51]. Similarly, analysis of 53 patients in the German skin cancer registry showed an ORR of 77.4% [52]. In Canada, a study of 36 patients with laBCC found a complete response rate of 50% and a partial response rate of 41.3%, with a median time to maximum response of 5.3 months; notably, 23.9% of patients became eligible for surgery after a median of 17.2 months [53]. A real-world multicenter cohort study further demonstrated that among 108 patients with laBCC and 13 with mBCC, vismodegib treatment was associated with overall survival rates of 86% at one year, 73% at two years, and 60% at three years, alongside a progression-free survival rate of 61% at one year [54]. In line with these findings, a nationwide retrospective study across 15 clinics reported a complete remission rate of 45.9% and an ORR of 77.5% [55]. Finally, a study of 61 French patients treated with vismodegib observed that no baseline factors predicted response; however, the presence of multiple BCCs was significantly associated with increased relapse risk (Hazard ratio/HR, 13.80), while longer treatment duration was linked to decreased relapse risk (HR, 0.95) [56].

Vismodegib has also demonstrated benefit in reducing tumor burden when used in combination with surgery. In patients with Basal Cell Nevus Syndrome, one study showed a significantly lower incidence of new surgically eligible BCCs compared with placebo [57]. Similarly, in a cohort of 15 large BCCs, a mean reduction of 27% in surgical defect size was observed after up to six months of therapy [58]. Despite these advantages, reports have also documented the development of resistance to vismodegib in some patients [59].

Following Vismodegib approval, sonidegib was subsequently approved in 2015 based on results from a randomized, double-blind phase II trial (BOLT) [60]. Like vismodegib, it is orally administered and targets SMO to inhibit HH signaling. Patients received either 200 mg or 800 mg of sonidegib once daily. At 42 months, the ORR among patients with laBCC treated with 200 mg was 56%, while those with mBCC achieved an ORR of 8%. The median duration of response for laBCC was 26.1 months [61]. When stratified by histologic subtype, patients with aggressive laBCC had a 42-month ORR of 59.5%, compared with 51.7% for nonaggressive subtypes. Among the aggressive variants, the highest ORRs were observed in infiltrative (200 mg, 51.6%) and morpheaform (200 mg, 50.0%) BCC [62].

Subsequent real-world data also showed promising results. A multicenter retrospective analysis of 82 real-world patients in Spain treated with sonidegib demonstrated that 81.7% experienced clinical improvement, including 29.3% with complete response and 52.4% with partial response. The median treatment duration in this cohort was six months, after which 48.8% of patients discontinued therapy. Notably, patients with prior vismodegib exposure and recurrent primary BCCs exhibited poorer responses to sonidegib [63]. Another recent retrospective study in Australia reported objective response rates of 92% for patients treated with sonidegib and 82% for those treated with vismodegib. This study also found a significantly higher incidence of dysgeusia with vismodegib compared to sonidegib (*p* = 0.0001), along with a shorter median time to onset of treatment-emergent adverse effects (TEAEs) in the vismodegib group [64].

While effective, HHIs are associated with class-specific adverse effects such as muscle cramps, alopecia, dysgeusia, weight loss, and fatigue [65]. These toxicities often limit long-term use, leading to treatment discontinuation in a substantial number of patients. Luckily, most adverse events were reversible after drug cessation [66]. Another clinical challenge is resistance to HHIs in advanced BCC. The most common mechanism involves acquired mutations in the SMO gene—notably D473H and W535L—which prevent drug binding while preserving downstream Hedgehog signaling activity [67]. Tumors may also activate noncanonical Hedgehog pathway signaling through Phosphatidylinositol 3-kinase/protein kinase B (PI3K/AKT) or RAS/MAPK pathways, promoting continued proliferation despite SMO inhibition [68]. Additionally, GLI2 amplification and loss of SUFU, a negative regulator of Hedgehog signaling, can confer downstream resistance to SMO blockade [69]. These mechanisms highlight the need for rational combination approaches to overcome pathway reactivation.

#### 2.3.2 Immune Checkpoint Inhibitors

In 2021, the FDA approved cemiplimab-rwlc, an anti–PD-1 monoclonal antibody, for the treatment of patients with laBCC or mBCC who have progressed on or are intolerant to HHIs. It is the first and currently the only FDA-approved ICI for these BCC indications, representing a critical option for patients who are unable to tolerate or do not respond to HHIs [45].

In the pivotal phase II clinical trial evaluated cemiplimab in patients with laBCC, patients were administering a 350 mg infusion every three weeks for up to 93 weeks or until treatment failure or intolerable side effects [70]. Among the 84 patients enrolled, the ORR was 31%, including 6% complete responses and 25% partial responses. The median time to response was 4.3 months, and 62% of patients discontinued therapy. Kaplan-Meier analysis estimated the probability of maintaining response at 85.2% at 12 months, while median progression-free survival was 19.3 months [70]. The leading cause of treatment discontinuation was disease progression (35%), followed by adverse events (16%) and patient choice (6%) [70]. In an extended follow-up, overall ORR increased slightly to 32.1%; specifically, ORR was 38.1% in patients intolerant to HHIs and 30.2% in those resistant to HHIs. Additionally, 70.4% of responders achieved greater than a 50% reduction in tumor size from baseline, and 59.5% remained progression-free for at least 182 days [71]. In metastatic BCC, a separate phase II trial involving 54 patients reported an ORR of 22%, with two complete responses and 10 partial responses, and a median time to response of three months [72]. Collectively, these findings demonstrate clinically meaningful benefit of cemiplimab in both laBCC and mBCC. The safety profile of cemiplimab is consistent with that of other immunotherapies. Grade 3 or 4 TEAEs were reported in 48% of patients, most commonly hypertension, colitis, and fatigue [70]. Serious TEAEs occurred in 35% of patients. Immune-related adverse events were observed in 25% of patients, including 10% with grade 3 severity [70]. Extended follow-up confirmed a consistent adverse event profile [71].

#### 2.3.3 Current and Emerging Combination Therapies

Recent data also suggest that combining Hedgehog pathway inhibition with immune checkpoint blockade may enhance therapeutic outcomes in advanced BCC [70,71]. The rationale behind this combination is because though HHIs can induce tumor regression by suppressing aberrant SMO signaling, resistance frequently develops through secondary SMO mutations or activation of noncanonical pathways. Additionally, prolonged HHI therapy may modify the tumor microenvironment, increasing MHC-I expression and enhancing T-cell infiltration, thereby potentially priming tumors for immune checkpoint blockade. Pirruccello and colleagues reported durable responses with combination therapy of vismodegib and cemiplimab in a patient with locally advance basosquamous carcinoma refractory to monotherapy [73]. In a recent multicenter retrospective cohort, sequential HHI followed by cemiplimab resulted in higher ORRs compared to cemiplimab alone, suggesting that HHI pretreatment may sensitize tumors to PD-1 inhibition [70,71]. Ongoing trials NCT04679480—anti-PD-1 antibody combined with pulsed HHI is formally evaluating this combination strategy [74]. Together, these findings support a growing paradigm in which vismodegib may be used as a cytoreductive and immune-priming agent prior to switching to or combining with cemiplimab in patients with aggressive or refractory disease.

Together, HHIs and ICIs represent complementary therapeutic strategies: HHIs are generally employed first-line for advanced BCC, while ICIs are considered in HHI-refractory or intolerant cases. Ongoing studies are exploring sequencing, other combination approaches, and predictive biomarkers to optimize patient selection and outcomes.

### 2.4 Neoadjuvant Targeted and Immunotherapies under Investigation for High-Risk BCC

Locoregional treatments for advanced laBCC often lead to substantial functional and aesthetic compromise, particularly for tumors arising in critical sites such as the nose and periorificial regions [75]. Neoadjuvant therapy is increasingly changing treatment paradigms in cutaneous oncology, including laBCC, by reducing tumor bulk prior to surgery. This approach aims to decrease the extent of surgical resection, lower postoperative morbidity, and enhance quality of life [76]. Early clinical trials of HHIs and ICIs in the neoadjuvant setting have reported promising results. Table 1 summarizes these trials.

**Table 1:** Clinical trials exploring neoadjuvant HHIs and ICIs in high-risk BCC

NCT	Title	Phase	Intervention	Status	Primary outcome/endpoint
NCT02667574 [68]	Study evaluating the interest of vismodegib as neo-adjuvant treatment of basal cell carcinoma (VISMONEO)	II	Vismodegib, surgery	Completed	‐ After a median treatment duration of 6 months, 44 of 55 patients (80%) reached a better surgical stage compared to baseline ‐ 27 patients (49%) obtained a clinical complete response
NCT03534947 [69]	A study to evaluate neoadjuvent sonidegib followed by surgery or imiquimod in the management of basal cell carcinoma (SONIB)	II	Sonidegib, imiquimod, surgery	Recruiting	‐ No reported result yet ‐ Primary endpoint is the tumor response at week 12
NCT05929664 [**79**]	Cemiplimab for the treatment of locally advanced head and neck basal cell carcinoma before surgery	II	Cemiplimab, surgery	Recruiting	‐ No reported result yet ‐ Primary endpoint is ORR and disease control rate by clinical and radiographic assessment
NCT04323202 [**81**]	Neoadjuvant-adjuvant pembrolizumab in resectable advanced basal cell carcinoma of head & neck	I	Pembrolizumab, surgery	Active, no recruiting	‐ No reported result yet ‐ Primary endpoint is pathologic response defined as change in tumor volume between baseline and surgery
NCT06624475 [**82**]	Neoadjuvant opdualag vs. nivolumab for resectable high-risk basal cell carcinoma	II	Nivolumab, relatlimab, surgery	Recruiting	‐ No reported result yet ‐ Primary endpoint is the pathologic response rate, which is defined as the proportion of patients achieving pathological complete response plus histologic major response

Note: HHIs, Hedgehog inhibitors; ICIs, Immune checkpoint inhibitors; BCC, Basal cell carcinoma.

The multicenter, open-label phase II VISMONEO trial (NCT02667574) evaluated neoadjuvant vismodegib in patients with facial aBCC considered inoperable or operable only with significant functional or aesthetic morbidity (Table 1) [77]. Participants received vismodegib 150 mg daily for 4 to 10 months, with surgical excision performed at the time of best observed response. The primary endpoint was the proportion of patients achieving surgical downstaging compared with baseline following neoadjuvant vismodegib. After a median treatment duration of six months, 44 of 55 patients (80%) demonstrated improved surgical staging, and 27 patients (49%) achieved a clinical complete response [77]. The non-randomized, open-label pilot study SONIB (NCT03534947) is evaluating the efficacy and safety of a 12-week course of neoadjuvant sonidegib 200 mg daily, followed by surgery or topical imiquimod, for BCCs located in cosmetically sensitive areas (Table 1) [78]. The primary endpoint is tumor response at week 12, assessed using optical coherence tomography (OCT) after completion of neoadjuvant therapy [78].

An ongoing single-center, single-arm, open-label phase II trial (NCT05929664) is evaluating neoadjuvant cemiplimab for laBCC of the head and neck prior to surgery (Table 1) [79]. Patients receive intravenous cemiplimab 350 mg every three weeks for up to six cycles, followed by surgical excision unless treatment is discontinued due to disease progression or unacceptable toxicity [79]. Another ongoing study evaluated pembrolizumab, which is a humanized monoclonal antibody targeting the PD-1 immune checkpoint [80]. A single-arm, open-label phase Ib trial (NCT04323202) is investigating neoadjuvant–adjuvant pembrolizumab in patients with resectable aBCC of the head and neck region (Table 1) [81]. Treatment consists of intravenous pembrolizumab 200 mg every three weeks for four cycles, followed by standard surgical resection and continuation of pembrolizumab in the adjuvant setting for a total of 17 cycles [81]. Lastly, a randomized, open-label, phase II clinical trial (NCT06624475) is currently investigating neoadjuvant nivolumab monotherapy or nivolumab and relatlimab combination in surgically resectable high-risk BCC patients (Table 1) [82]. Nivolumab is a humanized monoclonal antibody targeting the PD-1 immune checkpoint while relatlimab is a humanized monoclonal antibody that binds to the lymphocyte activation gene-3 (LAG3) receptor [80]. Both regimens are followed by surgical resection. In cases of disease progression, patients may proceed to surgical excision after two cycles of neoadjuvant immunotherapy [82].

These studies highlighted the recent advances of targeted and immunotherapy for the treatment of advanced BCC. In summary, across neoadjuvant studies in the setting of laBCC, HHIs remain the standard first-line therapies. HHIs induce rapid tumor shrinkage enabling surgical downstaging, particularly in cosmetically and functionally sensitive areas but are often followed by recurrence upon discontinuation which is drive by secondary SMO mutations, limiting long-term disease control [47,49]. In contrast, PD-1 blockade yields lower initial response rates but more durable responses and potential for long-term disease control, particularly in tumors with higher PD-L1 expression or evidence of T-cell infiltration [70]. Together, these patterns suggest a treatment sequencing model in which HHIs may be reserved for cytoreduction in surgically challenging tumors, whereas ICIs offer superior long-term control for biologically aggressive or immunogenic tumors.

## 3 Cutaneous Squamous Cell Carcinoma

### 3.1 Pathogenesis

cSCC arises from the malignant transformation of keratinocytes and represents approximately 20% of all nonmelanoma skin cancers [83]. The most well-established risk factor for cSCC is chronic exposure to UV radiation, particularly UVB. This is compounded by immune suppression, advanced age, fair skin, exposure to carcinogens (e.g., arsenic), and viral infections such as human papillomavirus (HPV) [84].

The pathogenesis of cSCC is driven by a complex network of deregulated signaling pathways. Both actinic keratosis (Aks) and cSCC lesions arise through a multistep process characterized by mutations in genes that regulate epidermal homeostasis, leading to uncontrolled proliferation of atypical keratinocytes. Notably, similar mutations are also detectable in normal keratinocytes, particularly in chronically sun-exposed skin [85]. This indicates that additional factors—such as epigenetic alterations, viral infections, and changes within the tumor microenvironment—contribute to the initiation and progression of cSCC [86]. At the molecular level, cSCCs harbor characteristic signature mutations in tumor suppressor genes such as TP53 and Cyclin-dependent kinase inhibitor 2A (CDKN2A) [87–90].

Activation of the catalytic subunit human telomerase reverse transcriptase (TERT) can occur through promoter (TERTp) mutations, which generate *de novo* binding sites for ETS family transcription factors. This activation extends telomere length and prevents senescence or apoptosis of mutated cells [91]. TERTp mutations with UV signatures have been identified in cSCC [92]. These mutations are more frequent in cSCC (50%) than in Bowen’s disease (20%), supporting their role in tumor progression [91]. Notably, TERTp mutations have been detected in 31.6% of cSCCs, primarily in recurrent and metastatic lesions [92]. However, additional studies are required to confirm the prognostic significance of TERTp mutations in cSCC.

Alterations in Neurogenic locus notch homolog protein 1/2 (NOTCH1/2), EGFR, and Rat sarcoma (RAS)-Mitogen-activated protein kinase kinase (MEK) pathways are also frequent in cSCC and contribute to cellular proliferation, survival, and differentiation [93–96]. Inactivating mutations of NOTCH1 have been identified in 42%–75% of aggressive or advanced cSCCs, while NOTCH2 mutations are reported in 18%–51% of cases [93,94]. An exome sequencing study published in 2012 further demonstrated NOTCH mutations in 82% of cSCCs and in 70% of normal sun-exposed skin, suggesting that alterations in NOTCH1 and NOTCH2 represent an early event in cSCC pathogenesis [93]. EGFR overexpression, indicative of constitutive pathway activation, has been reported in 43%–73% of cSCC cases and is associated with a more aggressive phenotype and poorer prognosis [95]. Importantly, EGFR activation suppresses p53 expression and subsequently downregulates NOTCH1 [92]. Lastly, activating mutations in HRAS have been identified in 3%–20% of cSCCs [90,96]. HRAS deletions have also been reported in approximately 10% of both primary and metastatic cSCCs [88,90]. Interestingly, HRAS mutations occur at a higher frequency in cSCC lesions that develop in melanoma patients treated with B-raf proto-oncogene (BRAF) inhibitors, a phenomenon likely explained by paradoxical MAPK pathway activation in keratinocytes harboring preexisting HRAS mutations [97].

Epigenetic alterations also play a key role in cSCC pathogenesis. DNA methylation, established by DNA methyltransferases, modifies cytosines at CpG sites, with cancer-associated changes including global hypomethylation and gene-specific hypermethylation [98]. UV irradiation induces large hypomethylated blocks in chronically sun-exposed skin, and methylation signatures are increasingly recognized as diagnostic and prognostic biomarkers [99]. In cSCC, promoter hypermethylation affects genes regulating the cell cycle (e.g., CDKN2A), apoptosis (e.g., Apoptosis-associated speck-like protein [ASC], G0/G1 switch gene 2 [G0S2], Death-associated protein kinase 1 [DAPK1]), Wnt signaling (Secreted frizzled-related proteins/SFRPs, Frizzled-related protein/FRZB), FOXE transcription factors, and adhesion molecules (e.g., Cadherin 1 [CDH1], Cadherin 13 [CDH13]) [100,101]. E-cadherin promoter hypermethylation was detected in 85% of cSCC, 50% of *in situ* cSCC, and 44% of AK, correlating with increased invasiveness and advanced disease [102,103]. Global methylation profiles distinguish AK/cSCC from normal skin and minimal signatures can stratify low- vs. high-risk cSCC, predicting overall survival [104,105].

Viral oncogenesis may also contribute, particularly in immunosuppressed populations. The detection of beta-HPV DNA in cSCC samples and HPV antibodies in patients suggests a role for beta-HPV in cSCC pathogenesis [106,107]. HPV-5 and HPV-8 are found in up to 90% of cases of epidermodysplasia verruciformis–associated cSCC [106], and beta-HPV is also implicated in cSCC among chronically immunosuppressed patients. Evidence indicates that beta-HPV contributes primarily to early carcinogenesis rather than progression, as viral load is higher in AKs than in cSCCs [108,109], and beta-HPV DNA is detected in only 9% of primary tumors and 13% of metastases [110]. The high prevalence of beta-HPV in normal skin highlights the importance of cofactors such as UV radiation, immunosuppression, chronic inflammation, and genetic susceptibility. Notably, concomitant seropositivity for mucosal HPV-16 and cutaneous HPV increases the risk of recurrent cSCC [107]. Thus, beta-HPV may act as a cofactor in the early stages of cSCC development by enhancing the effects of other carcinogenic drivers [106].

Overall, emerging data suggest that cSCC has one of the highest mutational burdens of any solid tumor, similar to melanoma [111]. This high mutation load promotes the formation of neoantigens that may be recognized by the immune system, providing a strong rationale for immunotherapy-based approaches.

### 3.2 Immune Landscape of Cutaneous Squamous Cell Carcinoma

The immune microenvironment in cSCC is dynamic and plays a dual role in both suppressing and promoting tumor development. On one hand, immune surveillance—particularly via cytotoxic T cells—can recognize neoantigens generated by the tumor’s high mutational burden. On the other hand, cSCC tumors develop several mechanisms of immune evasion to support progression involving other cell types.

Macrophages are abundant in cSCC compared with normal skin, where they function as tumor-associated macrophages (TAMs). These cells promote tumor growth and metastasis through the secretion of VEGF-C, which drives lymphangiogenesis, and MMPs, particularly MMP-9 and MMP-11, which remodel the extracellular matrix [112]. Within cSCC, TAMs display heterogeneous activation states, traditionally classified as M1 (pro-inflammatory, anti-tumor) or M2 (immunosuppressive, pro-tumor), though many exhibits mixed phenotypes [112]. Increased infiltration of M2-like TAMs correlates with poor prognosis [113], and their prevalence highlights TAMs as a potential immunotherapy target, where repolarization toward anti-tumor activity may slow disease progression.

T lymphocytes also play a critical role in cSCC immunity. Both conventional and organ transplant recipient (OTR)-associated cSCC harbor higher levels of CD3^+^ and CD8^+^ T-cells compared with normal skin, though these cells are typically concentrated in peritumoral rather than intratumoral regions [114]. Tregs, which suppress effector T-cell activity and IFN-γ production, are enriched in cSCC compared with normal skin and are associated with metastasis and poor prognosis [115,116]. While Treg numbers are similar in cSCC and OTR cSCC, the Treg-to-cytotoxic T-cell ratio is higher in OTR lesions, favoring immune evasion [116].

Functional deficiencies in NK cells are linked to increased cSCC incidence, and Nfil3^−/−^ knockout mouse models confirm their role in suppressing tumor initiation and growth [117–119]. However, NK cells isolated from cSCC tumors exhibit impaired cytotoxicity, reduced IFN-γ production, and lower secretion capacity, suggesting functional exhaustion within the tumor microenvironment [118]. Preclinical models demonstrate that adoptive transfer of healthy NK cells can restore tumor control, with both *in vitro* and *in vivo* studies showing reduced tumor burden and induction of apoptosis through suppression of Yes-1-associated transcription regulator (YAP1) and MEK signaling, highlighting their therapeutic potential [117].

Neutrophils, specifically tumor-associated neutrophils (TANs), are frequently found in both precancerous and invasive cSCC lesions, representing 30%–80% of infiltrating neutrophils [120]. Their role evolves across disease stages: in early lesions, they facilitate extracellular matrix remodeling and angiogenesis, whereas in invasive disease, they suppress anti-tumor immunity by inhibiting CD8^+^ T-cell activity and upregulating PD-L1 [120,121]. *In vivo* depletion of TAN enhances CD8^+^ T-cell proliferation and IFN-γ production, supporting their immunosuppressive role [120]. Clinically, elevated neutrophil counts correlate with increased tumor thickness and poor survival, suggesting their potential utility as prognostic markers [122].

Recent studies also highlight the prognostic role of tertiary lymphoid structures (TLS) in cSCC. TLS are organized aggregates of B cells, T cells, and dendritic cells that facilitate local antigen presentation and correlate with improved responses to ICIs [123]. Their presence reflects an inflamed, immunologically active tumor microenvironment, supporting TLS quantification as an emerging biomarker for immunotherapy response in cSCC.

Taken together, the immune landscape of cSCC is characterized by a complex interplay of immunosuppressive and anti-tumor populations. TAMs, Tregs, and TANs generally contribute to tumor progression and immune evasion, while cytotoxic T-cells and NK cells retain anti-tumor potential but are often functionally impaired. This balance highlights the importance of targeting immunosuppressive components of the tumor microenvironment while enhancing effector cell activity in future therapeutic strategies.

### 3.3 FDA-Approved Therapies for Cutaneous Squamous Cell Carcinoma

Historically, advanced cSCC was treated with chemotherapy or EGFR inhibitors off label with limited efficacy. However, recent advances have positioned ICIs as the standard of care for unresectable or metastatic disease.

#### 3.3.1 Immune Checkpoint Inhibitors

For advanced cSCCs not amenable to curative surgery or radiotherapy, immune checkpoint inhibition with cemiplimab, pembrolizumab, or cosibelimab is currently the preferred therapeutic option.

Cemiplimab, a high-affinity PD-1 monoclonal antibody, was the first ICI approved by both the United States FDA and the European Medicines Agency (EMA) for locally advanced and metastatic cSCC. It is administered intravenously every three weeks at a fixed dose of 350 mg [124]. Approval was based on the EMPOWER-CSCC-1 phase II trial, a multicohort study in which patients received cemiplimab either at 3 mg/kg every two weeks or 350 mg every three weeks. At 42.5 months, ORR among 193 patients treated with 3 mg/kg was 47%, with a median progression-free survival (PFS) of 26.0 months. In the 165 patients treated with 350 mg every three weeks, the ORR was 44%, and the median PFS was not reached. Grade 3–4 TEAEs occurred in approximately one-third of patients. EMPOWER-CSCC-1 remains the largest prospective study demonstrating long-term efficacy and safety of an ICI in advanced cSCC [124]. Supporting these findings, a retrospective multicenter Italian study of patients treated with cemiplimab at 17 referral centers reported an ORR of 58% and a disease control rate of 72% [125].

Importantly, the use of ICIs in solid-organ transplant recipients was recently evaluated in two promising studies. Hanna et al. evaluated modified doses of cemiplimab 350 mg IV once every 3 weeks for up to 2 years in kidney transplant recipients with advanced cSCC, aiming to reduce allograft rejection risk. Out of 12 treated patients, no kidney rejection or loss was observed. A response to cemiplimab was observed in 5 out of 11 evaluated patients with a median follow-up time of 6.8 months [126]. A multicenter phase 1/2 trial by Schenk and colleagues tested tacrolimus and prednisone with nivolumab in kidney transplant recipients with unresectable advanced cSCC or other skin cancers, with ipilimumab added at progression. All eight evaluable patients progressed on the initial regimen; six then received nivolumab plus ipilimumab, yielding two complete responses but four progressions, with median progression-free survival of 3 months and median overall survival of 9.1 months. Three patients experienced treatment-related allograft loss, two of whom showed donor-derived cell-free DNA (dd-cfDNA) elevations preceding serum creatinine increases, suggesting its potential as an early predictor of rejection [127].

Pembrolizumab has also shown robust and durable activity in locally advanced and relapsed cSCC [128]. In the phase II KEYNOTE-629 trial, patients received 200 mg every three weeks for up to 35 cycles. The study enrolled 159 patients, including 54 with locally advanced cSCC and 105 with relapsed or metastatic disease. In the locally advanced cohort, the ORR was 50.0% (95% CI, 36.1–63.9), including 16.7% complete responses and 33.3% partial responses. In the relapsed/metastatic cohort, the ORR was 35.2% (95% CI, 26.2–45.2), with 10.5% complete response and 24.8% partial response [128,129]. Building on this, Amatore et al. conducted a phase II single-arm neoadjuvant study of pembrolizumab in untreated, resectable locally advanced cSCC [130]. Eligible patients had AJCC/UICC ≥T3 or high-risk T2 tumors (≥2 cm, poorly differentiated histology, perineural invasion ≥ 0.1 mm, or invasion beyond fat) and/or nodal involvement [131]. Participants received two cycles of pembrolizumab (200 mg every three weeks) before surgery, followed by 15 cycles postoperatively (NCT04808999). Pathological complete response was achieved in 15 patients (57%), and in 14 of these, postoperative radiotherapy was de-escalated. Multiplex immunofluorescence identified PD-L1^+^/CD68^+^ cell interactions as biomarkers of response. The authors concluded that neoadjuvant pembrolizumab yielded a high pathological complete response rate, though confirmation in larger randomized phase III trials is needed [130].

Cosibelimab, another anti-PD-1 antibody, was more recently approved by the FDA for metastatic cSCC. Its registration was supported by a phase II trial in which patients received 800 mg intravenously every two weeks. The primary endpoint, ORR, was achieved in 37 of 78 participants (47.4%, 95% CI 36.0–59.1). The study demonstrated clinically meaningful responses with a manageable safety profile [132].

However, while cSCC is highly immunogenic and susceptible to ICIs, primary and acquired resistance to PD-1 inhibitors occurs in a significant subset of patients. A major mechanism is loss of antigen presentation through mutations or downregulation of β2-microglobulin (B2M) or MHC class I, which impairs CD8^+^ T-cell recognition [133]. Resistance may also develop through defects in interferon-γ (IFN-γ) signaling, especially Janus kinase 1 (JAK1) or Janus kinase 2 (JAK2) loss-of-function mutations, which blunt IFN-mediated tumor cell killing and reduce PD-L1 expression despite immune pressure [134].

#### 3.3.2 EGFR Inhibitors

Cetuximab, an EGFR inhibitor, has shown modest efficacy in advanced cSCC, particularly in patients unable to tolerate ICIs. It is not FDA-approved for this indication but is sometimes used off-label due to its approved use in SCC of the head and neck [135]. A retrospective multicenter study evaluated cetuximab and demonstrated moderate efficacy in patients with metastatic or locally advanced cSCC [136]. Reported outcomes included a median progression-free survival of 9.7 months and an overall survival of 17.5 months [136]. A phase II study of cetuximab as a first-line single-drug therapy in patients with unresectable cSCC showed a 69% disease control rate at 6 weeks [137]. However, although effective in subsets of patients, EGFR inhibitors are generally less durable in response and are associated with serious adverse events, including grade 4 infusion reactions and interstitial pneumopathy [137].

With the advent of immunotherapy, the role of EGFR-targeted agents has diminished. Still, cetuximab remains an important secondary-line option, particularly in cases of primary or acquired resistance to ICIs [138].

#### 3.3.3 Current and Emerging Combination Therapies

Combination strategies are also being explored to enhance responses in advanced cSCC, particularly for patients who progress on single-agent immunotherapy. The newly published prospective I-TACKLE study reported improved disease control and the ability to revert primary and acquired resistance when cetuximab was introduced following PD-1 blockade failure with pembrolizumab [139]. These findings support EGFR inhibition as a rational salvage or combination strategy by modulating tumor immunogenicity and overcoming adaptive resistance to PD-1 monotherapy.

There is also growing interest in combining cemiplimab with locoregional therapies that can enhance tumor antigen release. Retrospective analysis by Nardone et al. suggests that prior radiotherapy improves cemiplimab efficacy, while Bailly-Caille et al. reported concomitant administration of radiotherapy and cemiplimab enables earlier clinico-radiological response of 3 months, compared to 5.5 months in the cemiplimab-only group [140,141]. Similarly, photodynamic therapy (PDT) is emerging as a synergistic partner for immunotherapy in pre-clinical models of cSCC because PDT induces immunogenic cell death through ROS generation as well as damage-associated molecular patterns and promotes T-cell activation with enhanced antigen presentation [142]. However, the use of PDT in combination with ICIs has not been expanded to advanced cSCC in clinical trials. Integration of locoregional modalities such as PDT with systemic immunotherapy could represent an emerging direction for multimodal cutaneous oncology.

### 3.4 Neoadjuvant and Adjuvant Targeted and Immunotherapies under Investigation for High-Risk SCC

Active clinical trials for cSCC are expanding across several therapeutic domains: (1) the use of neoadjuvant ICIs to reduce tumor burden prior to surgery in high-risk resectable cSCC; (2) adjuvant ICIs to prevent recurrence after surgery in patients with high-risk features (e.g., perineural invasion, nodal involvement); and (3) intratumoral injections of ICIs to activate localized immune responses while minimizing systemic toxicity. Table 2 summarizes some of the ongoing trials.

**Table 2:** Clinical trials exploring neoadjuvant ICIs in high-risk cSCC

NCT	Title	Phase	Intervention	Status	Primary outcome/endpoint
NCT04154943 [**143**]	Study evaluating neoadjuvant plus adjuvant treatment with cemiplimab in stage III cSCC	II	Cemiplimab, surgery	Active, not recruiting	‐ No reported result yet ‐ Primary outcome is major pathological response rate, which is defined as <10% remaining viable tumor cells in resected primary tumor
NCT03969004 [**144**]	Study of adjuvant cemiplimab vs. placebo after surgery and radiation therapy in patients with high-risk cSCC	III	Cemiplimab, surgery, radiation	Active, not recruiting	‐ No reported result yet ‐ Primary outcome is disease-free survival, defined as time from randomization to the first documented disease recurrence or death due to any cause
NCT04808999 [**145**]	Neoadjuvant study of PD-1 inhibitor pembrolizumab in PD-1 naïve cSCC	II	Pembrolizumab, surgery	Active, not recruiting	‐ No reported result yet ‐ Primary endpoints are pathological response at time of surgery
NCT06288191 [**146**]	Neoadjuvant nivolumab and relatlimab in cSCC	II	Nivolumab, relatlimab, surgery	Recruiting	‐ No reported result yet ‐ Primary endpoint is pathologic response, defined as a change in tumor volume between baseline and surgery
NCT04620200 [**147**]	Neoadjuvant nivolumab or nivolumab with ipilimumab in advanced cSCC prior to surgery (MATISSE)	II	Nivolumab, ipilimumab, surgery	Completed	‐ No reported result yet ‐ Primary endpoint is the histopathological response rate which is the proportion of viable tumor cells left in the resected specimen at standard of care
NCT04710498 [**148**]	Neoadjuvant atezolizumab in surgically resectable advanced cSCC	II	Atezolizumab, surgery	Completed	‐ Results posted, not published ‐ 16 of 20 patients (80%) completed neoadjuvant therapy and were eligible for curative surgery ‐ Objective response rate: 5% complete response, 35% partial response, 50% stable disease, 5% disease progression ‐ Pathological response rate: 35% complete response, 20% major response, 40% no response
NCT03889912 [**149**]	Intralesional cemiplimab for adult patients with cSCC or BCC	I	Intralesional cemiplimab	Active, not recruiting	‐ No reported result yet ‐ Primary outcome measures are incidence, nature and severity of dose-limiting toxicities and treatment-emergent adverse events
NCT06014086 [**150**]	Intratumoral PH-762 for cutaneous carcinoma	I	Intralesional PH-762 (RNAi molecule targeting PD-1)	Recruiting	‐ No reported result yet ‐ Primary outcome measure is incidence, severity, seriousness and relatedness of all treatment-emergent adverse events

Note: ICIs, Immune checkpoint inhibitors; cSCC, Cutaneous squamous cell carcinoma; PD-1, Programmed cell death protein 1.

NCT04632433 is currently investigating the use of cemiplimab in neoadjuvant and adjuvant settings at a dosage of 350 mg every 3 weeks for two cycles prior to surgery in Stage III resectable cSCC. Postoperatively, adjuvant cemiplimab will be administered at a dosage of 350 mg every 3 weeks for one year [143]. NCT03969004 is a phase III multi-center international clinical trial that studies the use of adjuvant cemiplimab IV infusion vs. placebo after surgery and radiation therapy in patients with high-risk cSCC [144].

Several trials investigated other ICIs such as pembrolizumab, nivolumab, ipilimumab and atezolizumab. NCT04808999 is studying the neoadjuvant use of pembrolizumab in PD-1 naïve cSCC in a phase II trial. Patients will receive pembrolizumab peri-operatively for 6 weeks (200 mg every 3 weeks) prior to definitive surgery [145]. An Australian study, NCT06288191, is evaluating neoadjuvant therapy with the dual inhibition of PD-1 (nivolumab) and LAG-3 (relatlimab) in a cohort of treatment-naïve, resectable stage II to IV cSCC [146]. A recently completed phase II study in the Netherlands aimed to determine the histopathological response rate to neoadjuvant nivolumab and nivolumab plus ipilimumab at the time of surgery +/- radiation in patients with cSCC. Patients received either 2 courses of nivolumab 3 mg/kg or 2 courses of nivolumab plus 1 course of ipilimumab 1 mg/kg prior to standard of care [147]. Lastly, a study at Stanford evaluated neoadjuvant atezolizumab, a fully humanized monoclonal antibody against PD-L1, in 3 doses of 1200 mg prior to surgery in surgically resectable advanced cSCC. Preliminary results are summarized in Table 2 [148].

Few trials are currently investigating agents suitable for intralesional tumor injection. NCT03889912 is evaluating intratumoral injection of cemiplimab in cSCC and BCC in the US, Australia and the Netherlands [149]. Another trial evaluated the safety and tolerability of intratumoral injections of PH-762, a potent RNAi molecule targeting PD-1, in cSCC, melanoma or Merkel cell carcinoma of the skin in a phase I study [150].

Overall, these trials signify a promising future of the use of ICIs in advanced cSCC patients in neoadjuvant, adjuvant and intralesional settings. In summary, PD-1 blockade with cemiplimab or pembrolizumab consistently achieves durable responses (ORR 35%–50%) and has become the standard systemic therapy, demonstrating superior durability compared with EGFR inhibition, which historically produced short-lived responses and modest disease control [137,151–153]. In the neoadjuvant setting, PD-1 inhibitors have achieved major pathological response rates exceeding 50%, enabling curative surgery in borderline resectable cases and opening opportunities for postoperative radiation de-escalation [130]. By contrast, cetuximab and other EGFR inhibitors may be useful in ICI-refractory disease due to immunomodulatory effects but exhibit limited durability as monotherapy [154]. As resistance to PD-1 blockade emerges, rational combination strategies—such as PD-1 inhibitors with EGFR blockade, radiotherapy, or intralesional therapies like TLR agonists are all possibilities to enhance immune activation and broaden benefit.

## 4 Melanoma

### 4.1 Pathogenesis

Melanoma is a biologically heterogeneous malignancy arising from melanocytes, and its pathogenesis reflects both genetic alterations and environmental factors, particularly UV radiation exposure. Historically, melanoma was thought to follow a linear sequence from benign nevi to invasive and metastatic lesions [155]. However, recent evidence demonstrates a more complex and non-linear biology, with transformation potentially occurring directly from melanocyte stem cells or mature melanocytes. This contributes to marked heterogeneity in melanoma phenotypes, developmental states, and therapeutic responses [156].

UV radiation is one of the most important etiologic drivers in melanoma. Chronic UV exposure induces both quantitative and qualitative DNA damage, including C > T transitions at dipyrimidine sites and T > A transversions, collectively referred to as the “UV signature.” These mutations lead to a higher TMB, which correlates with cumulative sun damage (CSD) [156].

The molecular pathogenesis of melanoma involves alterations in several oncogenic pathways, usually in a mutually exclusive fashion. Low-CSD melanomas, such as superficial spreading melanoma, are most commonly driven by BRAF V600E mutations, often accompanied by alterations in TERT, CDKN2A, Phosphatase and tensin homolog (PTEN), or TP53 [157]. High-CSD subtypes, including lentigo maligna melanoma, show fewer BRAF V600E mutations but harbor NRAS (codon 61) and NF1 mutations, along with KIT alterations [158,159]. Desmoplastic melanomas are defined by an exceptionally high tumor mutational burden with frequent NF1 loss and gene amplifications, while spitzoid melanomas are characterized by HRAS mutations or oncogenic fusions involving Anaplastic lymphoma receptor tyrosine kinase **(**ALK), ROS proto-oncogene 1 (ROS1), Neurotrophic tyrosine receptor kinase (NTRK), BRAF, or Rearranged during transfection (RET) [157,160,161]. Acral and mucosal melanomas typically exhibit low mutational burden but frequent structural rearrangements and amplifications in TERT, Cyclin D1 (CCND1), Cyclin-dependent kinase 4 (CDK4), and KIT proto-oncogene (KIT), along with recurrent loss of CDKN2A and PTEN [160,162]. Melanomas arising in congenital nevi are commonly associated with NRAS or BRAF mutations, whereas those in blue nevi and uveal melanomas share Guanine nucleotide-binding protein Q polypeptide **(**GNAQ) and Guanine nucleotide-binding protein subunit alpha 11 **(**GNA11) mutations, often in combination with BAP1, SF3B1, or EIF1AX alterations [156,157,163]. Together, these pathways illustrate how melanoma arises from diverse genetic origins, with BRAF, NRAS, NF1, KIT, GNAQ, and GNA11 mutations representing the core oncogenic drivers across subtypes.

Beyond genetic mutations, epigenetic mechanisms—including DNA methylation, chromatin remodeling, and non-coding RNAs—play a role in melanoma pathogenesis. The tumor microenvironment further shapes disease behavior, with immune evasion facilitated by immunosuppressive cells and altered cytokine signaling. Melanomas with high TMB (especially CSD subtypes) are more likely to respond to immunotherapy, while low-TMB tumors (e.g., acral and mucosal) often show resistance [156].

### 4.2 Immune Landscape of Melanoma

Cutaneous melanoma is one of the most immunogenic human cancers, shaped profoundly by the tumor immune microenvironment. The high mutational burden, largely driven by UV exposure, generates a wealth of neoantigens that fuel robust immune recognition [164]. Yet, the immune system in melanoma plays a dual role—capable of mediating tumor regression, as evidenced in rare spontaneous regressions, but also co-opted by tumors to promote immune evasion and progression.

Tumor-infiltrating lymphocytes (TILs) are central to melanoma biology. High TIL density generally predicts favorable features and survival, though outcomes vary with composition [165]. CD8^+^ T cells are the main cytotoxic effectors, and tissue-resident CD103^+^CD8^+^ cells strongly predict survival and responsiveness to PD-1 blockade [166,167]. However, melanoma often induces exhaustion, limiting CD8^+^ activity. CD4^+^ T cells also influence outcomes: Th1 cells enhance cytotoxic responses and are favorable, while Th2 and some Treg subsets foster immune suppression [164]. Th17 cells can promote anti-tumor responses by recruiting effector cells. Tregs suppress cytotoxicity via CTLA-4, IL-10, and TGF-β; their impact is context-dependent, with higher numbers often linked to poor prognosis [168].

Among innate cells, NK cells detect MHC-I–deficient melanoma cells and mediate cytotoxicity, but are often scarce and dysfunctional; certain NK subsets nevertheless correlate with survival and improved responses to PD-1 therapy [169]. Dendritic cells (DCs), particularly conventional DC1s, drive CD8^+^ activation and correlate with better outcomes, whereas plasmacytoid DCs induce tolerance and predict worse prognosis [170,171]. Macrophages are usually skewed toward pro-tumor phenotypes, secreting IL-10, VEGF, and TGF-β, fostering angiogenesis and immunosuppression, and contributing to therapy resistance [172]. Neutrophils infiltrate melanoma but are generally associated with poor prognosis; TANs suppress CD8+ function and promote angiogenesis, though they may have context-dependent anti-tumor activity [171,173].

Last, melanoma exhibits complex immunologic resistance pathways due to its high tumor plasticity. PTEN loss leads to PI3K/AKT activation and promotes an immune-excluded phenotype, reducing T-cell infiltration and dampening ICI efficacy [174]. Mutations in the IFN-γ–JAK/STAT pathway (e.g., JAK1/JAK2) impair antigen presentation and drive acquired resistance to PD-1 therapy in metastatic melanoma [175]. Additionally, tumors upregulate alternative checkpoint receptors such as LAG-3, T cell immunoglobulin and mucin domain-containing protein 3 (TIM-3), and T-cell immunoreceptor with Ig and ITIM domains (TIGIT), which sustain T-cell exhaustion even under PD-1 blockade [176]. Some melanomas evade immunity through WNT/β-catenin–mediated exclusion of dendritic cells, resulting in T-cell desertification of the tumor microenvironment [177]. These insights have directly informed combination strategies in clinical trials.

Overall, melanoma progression reflects a balance between effector immune cells and immunosuppressive populations. The relative dominance of these cell types determines prognosis and therapeutic responsiveness. Checkpoint inhibitors have proven effective by reinvigorating exhausted T cells, but variability in immune composition and resistance patterns may explain the vast differences in patient outcomes. A deeper understanding of this landscape is critical for improving biomarkers and developing therapies that shift the tumor microenvironment toward sustained anti-tumor immunity.

### 4.3 Targeted Therapy for BRAF-Mutant Melanoma

In parallel with immunotherapy, targeted therapy remains the cornerstone of treatment for BRAF-mutant melanoma, which represents approximately 40–50% of cutaneous melanoma cases [178]. The phase III COMBI-AD trial established dabrafenib plus trametinib as a standard adjuvant regimen, achieving a 5-year RFS of 52% vs. 36% with placebo and a sustained overall survival benefit [179]. Similar adjuvant benefit was confirmed in the BRIM8 trial with vemurafenib and the COLUMBUS trial with combinational encorafenib/binimetinib programs, reinforcing the role of dual BRAF/MEK inhibition to delay recurrence after surgery [180,181]. Targeted therapy provides rapid disease control and high response rates, making it particularly valuable in symptomatic, high-tumor burden, or rapidly progressive disease, where time to response is critical [179,180]. However, resistance commonly develops through MAPK pathway reactivation, secondary NRAS mutations, BRAF splice variants, or activation of the PI3K-AKT pathway, which limits the durability of targeted therapy compared with immunotherapy [182]. As a result, treatment sequencing strategies are evolving: many centers favor an immunotherapy-first approach due to superior long-term survival outcomes, while BRAF/MEK inhibitors are prioritized in scenarios requiring rapid cytoreduction, management of steroid-dependent brain metastases, or intolerance to checkpoint inhibitors [183,184]. Recent trials such as DREAMseq and SECOMBIT support this sequencing rationale, demonstrating improved survival when PD-1–based therapy precedes BRAF/MEK inhibition rather than the reverse, underscoring the importance of strategic integration of targeted therapy in precision melanoma management [185,186].

### 4.4 Current and Emerging Immunotherapies for Melanoma

**Figure 1:**
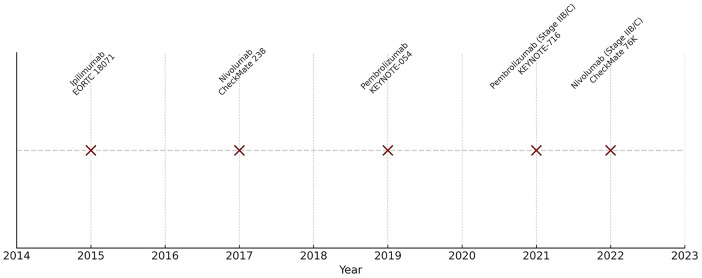
Timeline of FDA approvals for immunotherapies in melanoma with pivotal trials

Immunotherapy has revolutionized melanoma treatment, shifting the paradigm from high-dose interferon-α and interleukin-2 to ICIs, which now constitute the standard of care in both adjuvant and neoadjuvant settings. Several PD-1 and CTLA-4–targeting agents have secured FDA approval based on robust clinical evidence (Fig. 1). Additional evidence and ongoing trials are also summarized below.

#### 4.4.1 Ipilimumab—EORTC 18071

Ipilimumab was the first ICI approved in melanoma, targeting CTLA-4 [187]. The EORTC 18071 trial enrolled 951 patients with resected stage III melanoma randomized to ipilimumab vs. placebo. At five years, ipilimumab improved recurrence-free survival (RFS) (40.8% vs. 30.3%) and overall survival (OS) (65.4% vs. 54.4%). However, its use was limited by high toxicity, with up to 40% grade 3–4 TEAEs, leading to discontinuation in many patients [187]. Although it established the principle of adjuvant immunotherapy, ipilimumab has largely been supplanted by PD-1 inhibitors.

#### 4.4.2 Nivolumab—CheckMate 238

Nivolumab emerged as a superior alternative in recent years [188,189]. In the CheckMate 238 phase III trial, 906 patients with resected stage IIIB, IIIC, or IV melanoma were randomized to nivolumab (3 mg/kg q2w) or ipilimumab. Nivolumab achieved significantly higher 12-month RFS (70.5% vs. 60.8%) with an HR of 0.65, leading to FDA approval in 2017. At five years, RFS remained superior with nivolumab (50% vs. 39%; HR 0.72), though OS was not significantly different, likely due to crossover and effective salvage therapies. Importantly, nivolumab demonstrated a better safety profile than ipilimumab, with fewer high-grade immune-related toxicities [188,189].

#### 4.4.3 Pembrolizumab—KEYNOTE-054

Pembrolizumab further cemented PD-1 inhibition in the adjuvant setting [190,191]. In KEYNOTE-054, 1019 patients with completely resected stage III melanoma were randomized to pembrolizumab (200 mg q3w for 1 year) or placebo. At 12 months, RFS was significantly longer with pembrolizumab (75.4% vs. 61.0%; HR 0.57). Long-term follow-up confirmed durable benefit: at five years, RFS was 55.4% with pembrolizumab vs. 38.3% with placebo (HR 0.61). This trial supported pembrolizumab’s FDA approval in 2019 as adjuvant therapy for stage III melanoma [190,191].

#### 4.4.4 Pembrolizumab vs. Interferon or Ipilimumab—SWOG 1404

The SWOG 1404 intergroup trial compared pembrolizumab to either high-dose interferon-α or ipilimumab in 1301 patients with stage III/IV melanoma. With a median follow-up of 47.5 months, pembrolizumab demonstrated longer RFS (HR 0.77), though no OS difference was detected, likely due to subsequent anti-PD-1 use in control arms. This trial reinforced pembrolizumab as superior to prior standards [192].

#### 4.4.5 Combination Therapy—IMMUNED, CheckMate 915, and Beyond

Given the success of ipilimumab–nivolumab in metastatic melanoma, its use was tested in resected disease. The IMMUNED phase II trial randomized 167 patients with stage IV melanoma rendered disease-free to nivolumab + ipilimumab, nivolumab alone, or placebo. Combination therapy substantially improved RFS (HR 0.25) and OS (HR 0.41) compared to placebo, suggesting a role in high-risk patients [193,194].

However, the larger CheckMate 915 phase III trial in 1833 patients with stage IIIB–IV melanoma failed to demonstrate additional benefit of nivolumab + low-dose ipilimumab compared to nivolumab monotherapy (24-month RFS ~65% in both arms) [195]. However, dosing of nivolumab and ipilimumab were significantly different in this trial compared to IMMUNED. This result highlights dosing strategies and trial design as critical factors.

Despite setbacks, combination targeted-therapy/immunotherapy has emerged as a strategy to overcome resistance to single-agent PD-1 blockade in melanoma. Dual immune checkpoint inhibition with nivolumab plus relatlimab, an anti–LAG-3 antibody, has demonstrated improved PFS compared with nivolumab alone in the phase II/III RELATIVITY-047 trial and is now FDA-approved for advanced melanoma, highlighting LAG-3 as a clinically actionable exhaustion pathway [196]. Beyond LAG-3, additional inhibitory receptors are under investigation to enhance T-cell reinvigoration. Combinations of PD-1 inhibitors with intratumoral toll-like receptor 9 (TLR9) agonists to promote intratumoral dendritic cell activation and enhance antigen presentation showed promising results, with 25% durable responses in PD-1 therapy-refractory patients [197]. Together, these approaches reflect a shift toward rational immune modulation to broaden the depth and durability of responses in advanced melanoma.

#### 4.4.6 Expanding Indications for Stage II Melanoma—KEYNOTE-716 and CheckMate 76K

Historically, adjuvant therapy was reserved for stage III disease. Two key trials extended PD-1 blockade into stage II.

KEYNOTE-716 randomized 976 patients with resected stage IIB/C melanoma to pembrolizumab or placebo. At 18 months, RFS was 86% in patients who received pembrolizumab adjuvant therapy vs. 77% in patients who received placebo (HR 0.61). At 24 months, pembrolizumab maintained a 6% absolute benefit over placebo [198,199]. This supports the expanding indication of pembrolizumab for stage IIB/C melanoma based on improved RFS. CheckMate 76K tested nivolumab in 790 patients with resected stage IIB/C melanoma, showing 12-month RFS of 89% vs. 79% with placebo (HR 0.42). Grade 3/4 adverse events occurred in 10.3% of patients, indicating that nivolumab is a well-tolerated regimen for stage IIB/C resected melanoma [200].

These results led to FDA approvals for both pembrolizumab and nivolumab in stage IIB/C disease. These approvals reflect recognition of recurrence risk in stage II disease, comparable to some stage III subgroups.

#### 4.4.7 Neoadjuvant Approaches—SWOG S1801 and Beyond

The SWOG S1801 phase II trial shifted the paradigm, demonstrating that perioperative pembrolizumab (3 doses pre-surgery + adjuvant continuation) improved 2-year event-free survival compared to adjuvant-only therapy (72% vs. 49%; HR 0.58). Pathologic complete response was achieved in 21% of patients [201]. These findings support neoadjuvant strategies as potentially superior, though not yet FDA-approved. Several completed and ongoing trials (e.g., NADINA, OPACIN-NEO, PRADO) are evaluating optimal combinations and sequencing for neoadjuvant ipilimumab and nivolumab [202–204]. Lastly, Amaria et al. evaluated combinational neoadjuvant relatlimab and nivolumab in patients with unresectable advanced melanoma, which resulted in a 57% pathological complete response and 70% overall pathological response rate among 30 patients treated. No grade 3–4 immune-related adverse events were observed in the neoadjuvant setting [205]. Long-term survival analysis showed that at 4 years from the start of neoadjuvant treatment, 80% of patients remain event-free, including 95% of patients who achieved a major pathologic response [206]. This provided additional evidence for a novel combination of ICIs for the treatment of melanoma.

Interestingly, response rates to PD-1 blockade vary across tumor types: melanoma demonstrates the highest ORR (40–60%) due to its high mutational burden and immune infiltration, followed by cSCC (35%–50%) and BCC (~30%) [70,151,188]. However, melanoma patients also experience the highest incidence of grade ≥ 3 immune-related adverse events (up to 20%–30%), whereas cSCC and BCC exhibit lower rates (<15%) [59,128,207]. These distinctions underscore the differential immunogenicity and microenvironmental constraints across cutaneous malignancies.

Overall, the repertoire of available ICIs for the treatment of high-risk melanoma is steadily expanding and melanoma remains the prototype of successful cancer immunotherapy. Anti–PD-1 monotherapy (nivolumab or pembrolizumab) achieves durable survival in ~40%–45% of advanced cases, while dual checkpoint blockade (nivolumab + ipilimumab) improves depth of response at the cost of increased toxicity [188,189,208]. The recent approval of nivolumab + relatlimab (anti–LAG-3) provides a less toxic alternative to CTLA-4–based combination therapy, marking a clinically meaningful advance in overcoming T-cell exhaustion [196]. In patients with BRAF-mutant melanoma, adjuvant BRAF/MEK inhibition (dabrafenib + trametinib) improves relapse-free survival and competes with anti–PD-1 agents in high-risk resected disease, creating a critical need for sequencing strategies that balance rapid cytoreduction with long-term immune durability [179]. Neoadjuvant immunotherapy has demonstrated superior event-free survival compared with adjuvant-only approaches, reshaping treatment algorithms for stage III melanoma [201]. Yet resistance persists, driven by loss of interferon signaling, PTEN loss, and immune exclusion through WNT/β-catenin signaling, motivating combination trials targeting LAG-3, TIGIT, and TLR agonists to extend benefit to non-responders [174,175,197].

## 5 Emerging Biomarkers

Excitingly, new biomarkers are beginning to guide response prediction and treatment selection across cutaneous malignancies. In BCC, potential biomarkers include TMB, PD-L1 expression, and restoration of antigen-presenting capabilities, particularly MHC-I upregulation following HHI exposure, which may predict enhanced responsiveness to PD-1 blockade [209,210]. In cSCC, biomarkers such as high TMB, PD-L1 expression, and IFN-γ gene signatures correlate with favorable responses to immunotherapy, reflecting a pre-existing T cell-inflamed tumor microenvironment. Conversely, loss of HLA class I or β2-microglobulin may indicate immune evasion and resistance [96,151,211]. In melanoma, several biomarkers have stronger clinical validation, including TMB, TIL density, PD-L1 expression, and BRAF mutation status, which help guide the use of targeted therapy [212,213]. Beyond tumor-intrinsic biomarkers, immune-related biomarkers are gaining interest. For example. LAG-3 expression and T-cell exhaustion signatures are being explored as indicators of potential benefit from dual checkpoint inhibition, while circulating cell-free DNA kinetics offer a promising non-invasive method to monitor minimal residual disease and early treatment response across melanoma and nonmelanoma skin cancers [196,214,215].

## 6 Conclusion

The therapeutic armamentarium for cutaneous malignancies has expanded dramatically with the advent of immunotherapy and targeted agents. Once considered disparate in prognosis and biology, BCC, cSCC, and melanoma now share a growing number of treatment modalities. This convergence is driven by deeper understanding of tumor immunobiology and the successful translation of checkpoint inhibitors across cancer types.

BCC remains unique due to its Hedgehog dependency and immunosuppressive microenvironment, yet its responsiveness to PD-1 blockade in select cases opens doors for combinatorial approaches. cSCC, with its UV-driven mutations and emerging role for neoadjuvant immunotherapy, reflects an evolving model of integrated oncologic and dermatologic care. Melanoma continues to lead the field with refined regimens, adjuvant and neoadjuvant applications, and personalized strategies based on BRAF status and immunogenicity.

Ultimately, the future of skin cancer management will lie in precision immunotherapy—tailoring treatments to molecular features, immune contexture, and patient-specific factors to optimize outcomes and minimize toxicity.

## Data Availability

Not applicable.
